# Physical Activity Habit Automaticity, Psychological Distress, and Quality of Life Among Chinese University Students: A Three-Wave Longitudinal Moderated Mediation Study

**DOI:** 10.3390/healthcare14131863

**Published:** 2026-06-26

**Authors:** Yanli Tan, Shiqi Liu, Liuhong Zang

**Affiliations:** School of Physical Education, Xinjiang Normal University, Urumqi 830054, China

**Keywords:** physical activity habit automaticity, exercise identity, psychological distress, quality of life, university students, moderated mediation

## Abstract

Background: Physical activity is widely linked to better mental health and quality of life in university students, but less is known about whether the automatic, habit-like quality of physical activity is prospectively associated with quality of life through psychological distress and whether this indirect association depends on exercise identity. The objective of this study was to test a three-wave longitudinal moderated mediation model linking T1 physical activity habit automaticity, T2 psychological distress, T3 quality of life, and T1 exercise identity. Methods: A three-wave longitudinal survey was conducted among Chinese university students in 2026. After data cleaning and anonymous matching across waves, the final analytic sample included 1024 students, of whom 58.98% were female. T1 physical activity habit automaticity was assessed using the Self-Report Behavioral Automaticity Index, T1 exercise identity using the Exercise Identity Scale, T2 psychological distress using the Patient Health Questionnaire-4, and T3 quality of life using the EUROHIS-QOL 8-item index. PROCESS Model 4 and Model 7 were estimated with 5000 bootstrap samples, controlling for sex, age, grade, physical activity frequency, sleep quality, baseline psychological distress, and baseline quality of life. Results: In the baseline-adjusted mediation model, T1 physical activity habit automaticity was negatively associated with T2 psychological distress (B = −0.503, *p* < 0.001), and T2 psychological distress was negatively associated with T3 quality of life (B = −0.086, *p* < 0.001). The indirect effect was significant (effect = 0.043, 95% CI [0.028, 0.059]), with a mediated proportion of 30.9%, while the direct effect remained significant. In the moderated mediation model, the habit automaticity × exercise identity interaction significantly predicted T2 psychological distress (B = −0.317, *p* < 0.001, ΔR^2^ = 0.008). Conditional indirect effects were significant at mean and high levels of exercise identity, and the index of moderated mediation was significant (index = 0.027, 95% CI [0.012, 0.043]). Conclusions: Physical activity habit automaticity was prospectively associated with higher quality of life partly through lower psychological distress, and this indirect association was more pronounced among students with stronger exercise identity. The findings highlight habit formation and exercise identity as potentially relevant targets for campus health promotion, although causal conclusions remain limited by the observational design.

## 1. Introduction

University life is a developmental period in which academic demands, social transitions, and future-oriented uncertainty often coincide with emerging mental health problems. Large-scale international work has shown that mental disorders are common among college students, and such problems may interfere with functioning during a stage in which educational and social trajectories are being consolidated [1]. Evidence from college freshmen further indicates that psychological problems are not only prevalent but also related to academic functioning and adjustment [2]. From a life-course perspective, the typical age of university students overlaps with the onset period of many common mental disorders, making preventive health strategies particularly relevant in this population [3]. Anxiety and depressive symptoms have also been widely documented in student populations, including in medical and general university contexts [4,5]. Beyond symptom burden, quality of life offers a broader health-oriented outcome because it captures how students evaluate their physical, psychological, social, and environmental circumstances [6,7].

Physical activity is a plausible non-pharmacological correlate of student mental health because it is embedded in daily routines, requires limited specialized infrastructure, and can be promoted through campus environments. Global public health guidelines emphasize the importance of regular physical activity for health across the life course [8]. Meta-analytic and review evidence has linked physical activity with lower risks of depression and anxiety, as well as with better psychological functioning [9,10]. Similar evidence has been reported for anxiety-related outcomes and broader well-being, although effect sizes vary by population, measurement, and study design [11,12]. Recent systematic evidence among college students has further reported a weak but positive association between physical activity and overall quality of life [13]. Mechanistic accounts of the physical activity-mental health relationship emphasize biological, affective, cognitive, and social processes, including mood regulation, mastery experiences, sleep, and social participation [14]. At the same time, insufficient activity remains a public health concern among college-aged students, suggesting that sustainable physical activity engagement should be studied not only as a matter of frequency but also as a matter of behavioral maintenance [15].

Most student health studies measure physical activity as volume, frequency, or intensity. These indicators are important, but they do not capture whether physical activity has become an efficient, cue-driven behavior that can be initiated with minimal conscious deliberation. Habit theory proposes that repeated behavior in stable contexts can gradually become automatic, allowing behavior to proceed without continuous reflective control [16,17]. The Self-Report Habit Index and its automaticity subscale provide the measurement foundation for assessing this automatic quality of behavior [18,19]. A key implication is that habit automaticity differs from simple activity frequency: two students may report similar weekly activity, but only one may experience physical activity as a routine that is easy to initiate under familiar cues. Health-behavior research has therefore emphasized habit as a process supporting sustained behavior rather than a synonym for behavioral quantity [20,21]. Self-regulatory and dual-process accounts make this perspective especially relevant to university students, whose academic and social pressures may reduce available self-regulatory resources [22,23]. Physical activity theories further suggest that intention-based exercise may be difficult to maintain when students encounter competing demands, making automatic routines potentially important [24,25].

Psychological distress may be a proximal pathway linking physical activity habit automaticity with later quality of life. In this study, psychological distress refers to anxiety and depressive symptom burden assessed by the Patient Health Questionnaire-4 (PHQ-4), a short screening instrument integrating anxiety and depression symptoms [26,27]. Recent Chinese longitudinal evidence supports the psychometric suitability of the PHQ-4 for repeated assessment in student-related samples [28]. Anxiety and depressive symptoms are closely related to reduced energy, diminished self-esteem, impaired daily functioning, and poorer interpersonal experiences, all of which are central to health-related quality of life. The PHQ family and related anxiety measures have strong evidence as brief indicators of emotional symptom burden [29,30]. Quality of life was assessed using the EUROHIS-QOL 8-item index, a brief cross-cultural instrument derived from the WHOQOL framework and designed for public health applications [31,32]. Related WHOQOL-BREF evidence has also supported quality-of-life assessment across diverse health conditions [33]. Conceptually, if habitually automated physical activity is associated with lower subsequent distress, lower distress may in turn be associated with better later quality of life.

Exercise identity may mark a boundary condition under which the association between habit automaticity and psychological distress becomes more pronounced. Exercise identity refers to the extent to which a person incorporates the role of being an exerciser into the self-concept, and the Exercise Identity Scale was developed to capture this self-definitional aspect of exercise participation [34]. Identity theory suggests that behavior aligned with a salient role identity is more likely to be self-verifying and psychologically coherent [35,36]. Related work distinguishing identity theory from social identity theory further indicates that role-based self-meanings can guide behavior and affective responses [37]. In the physical activity domain, students with stronger exercise identity may interpret automatic physical activity not merely as a routine but as behavior consistent with who they are. When habitual physical activity aligns with a salient exercise identity, the behavior may be experienced as more self-consistent, potentially amplifying its association with lower emotional distress. Earlier identity research in exercise settings supports the relevance of self-definition to exercise-related responses [38,39]. Meta-analytic and theory-oriented work also shows that physical activity identity is closely linked to activity behavior and may be strengthened through targeted strategies [40,41]. Chinese validation evidence further supports the use of exercise identity measurement in college student samples [42].

The objective of the present study was to test a three-wave longitudinal conditional process model among Chinese university students. Specifically, T1 physical activity habit automaticity was specified as the predictor (X), T2 psychological distress as the mediator (M), T3 quality of life as the outcome (Y), and T1 exercise identity as a first-stage moderator (W). This design allowed us to examine whether habit automaticity was prospectively associated with later quality of life, whether psychological distress statistically accounted for this association, and whether the first-stage pathway was stronger among students with a stronger exercise identity. This approach follows contemporary recommendations for testing indirect and conditional indirect associations while maintaining temporal separation between predictor, mediator, and outcome [43,44]. Regression-based mediation and moderation frameworks also provide implementation guidance for estimating such models with observed variables [45,46]. Because indirect effects and interactions are often small, sufficient sample size is important for stable inference [47]. Bootstrap confidence intervals and attention to interaction effect size are also recommended when interpreting conditional process models [48,49]. To reduce common method concerns, analyses used temporal separation and controlled for baseline values where available [50]. Attrition and power-related considerations were also addressed in the analytic workflow [51,52]. The study tested five hypotheses: H1, T1 habit automaticity would be positively associated with T3 quality of life; H2, T1 habit automaticity would be negatively associated with T2 psychological distress; H3, T2 psychological distress would mediate the association between T1 habit automaticity and T3 quality of life; H4, exercise identity would moderate the T1 habit automaticity to T2 psychological distress path; and H5, the indirect association would vary by exercise identity.

## 2. Materials and Methods

### 2.1. Study Design and Participants

This study used a three-wave longitudinal survey design. Data were collected in 2026 across three survey waves. The adjacent waves were separated by approximately five weeks, and the full follow-up period covered approximately ten to eleven weeks. Participants were Chinese university students recruited through convenience sampling from campus- and course-related channels. Eligible participants were students aged 18 years or older who were enrolled in undergraduate or postgraduate study and were able to complete the Chinese questionnaire independently. Online survey links were distributed through campus- and course-related channels to increase accessibility. Anonymous matching codes were used to link responses across waves without collecting names or student identification numbers.

At T1, 1532 questionnaires were returned, and 1351 were initially valid after preliminary data screening. After removal of baseline records with invalid, duplicated, or unmatchable anonymous codes, 1282 T1 records were eligible for matching-code standardization and longitudinal linkage. At T2, 1444 questionnaires were returned, and 1329 were valid. At T3, 1270 questionnaires were returned, and 1089 were valid. After matching responses across all three waves and excluding invalid or unmatched records, the final analytic sample consisted of 1024 participants. The final sample included male and female students from first year through postgraduate study, with ages ranging from 18 to 27 years.

### 2.2. Measures

No commercial physical measuring instruments were used in this study; all variables were assessed using self-report questionnaire scales administered online.

#### 2.2.1. Physical Activity Habit Automaticity

Physical activity habit automaticity was measured at T1 using the four-item Self-Report Behavioral Automaticity Index (SRBAI). Items were scored on a 7-point scale, with higher scores indicating stronger automaticity of physical activity. The item mean was used in the analyses.

#### 2.2.2. Exercise Identity

Exercise identity was measured at T1 using the nine-item Exercise Identity Scale (EIS). Items were scored on a 7-point scale, with higher scores indicating stronger incorporation of exercise into the self-concept. The item mean was used in the analyses.

#### 2.2.3. Psychological Distress

Psychological distress was assessed at T1 and T2 using the Patient Health Questionnaire-4 (PHQ-4), which includes two anxiety items and two depressive symptom items scored from 0 to 3. Total scores range from 0 to 12, with higher scores indicating greater distress. T1 psychological distress was used as a baseline covariate, and T2 psychological distress was specified as the mediator.

#### 2.2.4. Quality of Life

Quality of life was assessed at T1 and T3 using the EUROHIS-QOL 8-item index. Items were scored from 1 to 5, and the item mean was used, with higher scores indicating better quality of life. T1 quality of life was used as a baseline covariate, and T3 quality of life was specified as the outcome.

#### 2.2.5. Covariates

Covariates included sex, age, grade, physical activity frequency, sleep quality, baseline psychological distress, and baseline quality of life. Physical activity frequency was assessed by asking how many days in the past seven days participants engaged in at least 30 min of moderate-or-higher intensity physical activity. Sleep quality was assessed with one item referring to the previous seven days, scored from 1 (very poor) to 5 (very good). In the PROCESS models, covariates were entered into the mediator and outcome equations to adjust the estimated indirect and conditional indirect associations.

### 2.3. Data Cleaning and Statistical Analysis

Data were cleaned according to predefined criteria. Questionnaires were excluded if they had substantial missing data, failed the attention-check item, were completed in an unrealistically short time, or contained implausible demographic information. Duplicate submissions were identified using anonymous matching codes and response timestamps. For repeated submissions within the same wave, only one valid response was retained based on completion status, attention-check performance, plausible response time, and submission timestamp. Matching codes were standardized by removing spaces and unifying letter case. Records with contradictory demographic information across waves were not matched.

Descriptive statistics, internal consistency coefficients, skewness, kurtosis, attrition analyses, Harman’s single-factor test, and Pearson correlations were calculated before model testing. Pearson correlation coefficients were interpreted using Cohen’s conventional guidelines, with absolute values around 0.10, 0.30, and 0.50 considered weak, moderate, and strong associations, respectively. PROCESS Model 4 was used to test the longitudinal indirect association, and PROCESS Model 7 was used to test first-stage moderated mediation. SRBAI and EIS were mean-centered before computing the interaction term. Conditional effects were estimated at low, mean, and high levels of exercise identity, corresponding to one standard deviation below the mean (−1 SD), the sample mean, and one standard deviation above the mean (+1 SD). Indirect effects, conditional indirect effects, and the index of moderated mediation were estimated with 5000 bootstrap samples and 95% confidence intervals. The mediated proportion (PM) was calculated as the indirect effect divided by the total effect in the simple mediation model. In the moderated mediation model, conditional mediated proportions were calculated by dividing each conditional indirect effect by the total effect and were interpreted descriptively because conditional indirect effects vary across levels of the moderator. Statistical significance was evaluated at *p* < 0.05. Because the study was observational, the term mediation is used in a statistical and temporally separated sense rather than as evidence of definitive causality. Analyses were performed using IBM SPSS Statistics version 26.0 (IBM Corp., Armonk, NY, USA) with PROCESS macro version 4.2.

### 2.4. Ethics Approval

The study was approved by the Ethics Committee of Xinjiang Normal University (Approval No. XJNU2026LLSC53). All participants provided informed consent before completing the online questionnaire. Participation was voluntary, and responses were anonymized using matching codes.

## 3. Results

### 3.1. Sample Flow, Participant Characteristics, and Attrition

The final three-wave matched analytic sample included 1024 students. Women accounted for 58.98% of the sample, and the mean age was 19.93 years (SD = 1.60). Most participants were undergraduates, with first-, second-, and third-year students forming the largest groups. The mean physical activity frequency was 2.63 days per week, and the mean sleep quality score was 3.29. As shown in Table 1, the final analytic sample was obtained after sequential screening, matching-code standardization, and three-wave linkage. Table 2 indicates that the sample covered both sexes and multiple academic grades, supporting the suitability of the data for testing the proposed longitudinal conditional process model. Attrition analyses did not indicate systematic baseline differences between completers and participants lost to follow-up.

### 3.2. Reliability, Distributional Characteristics, and Common Method Bias

All multi-item scales showed acceptable to excellent internal consistency, with Cronbach’s alpha values ranging from 0.833 to 0.932 (Table 3). The descriptive statistics also indicated adequate variability in the main study variables. Skewness and kurtosis values were within conventional acceptable ranges for parametric analyses. Harman’s single-factor test indicated that the first unrotated factor accounted for 34.85% of the total variance, below the commonly used 40% threshold. This suggests that common method bias was unlikely to be severe, although shared method variance cannot be completely ruled out because all measures were self-reported.

### 3.3. Correlation Analysis

The correlation matrix supported the expected directions of association (Table 4). T1 physical activity habit automaticity showed a moderate negative correlation with T2 psychological distress and a moderate positive correlation with T3 quality of life. T2 psychological distress was strongly and negatively correlated with T3 quality of life. Exercise identity was moderately and positively correlated with habit automaticity and was weakly to moderately correlated with psychological distress and quality of life. These bivariate patterns provided preliminary support for the hypothesized mediation and moderated mediation analyses, while the magnitude of correlations also indicated that the constructs were related but not redundant.

### 3.4. Longitudinal Mediation Analysis

**Figure 1:**
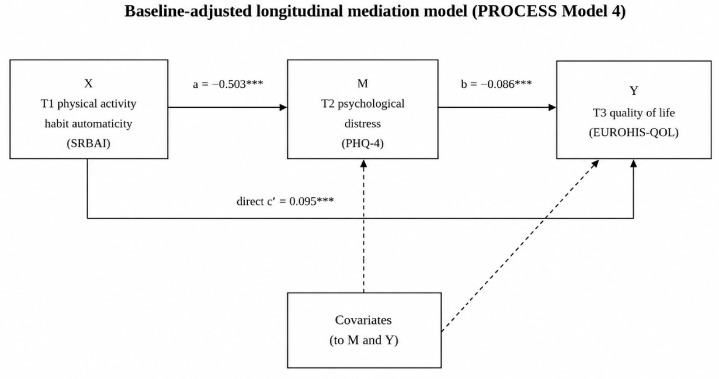
Baseline-adjusted longitudinal mediation model (PROCESS Model 4). Note. X = T1 physical activity habit automaticity; M = T2 psychological distress; Y = T3 quality of life. Solid arrows indicate hypothesized direct paths, and dashed arrows indicate covariate-adjustment components. Covariates were entered into the mediator and outcome equations. Values are unstandardized coefficients. The indirect effect was 0.043, 95% CI [0.028, 0.059], and the mediated proportion was 30.9%. *** *p* < 0.001.

PROCESS Model 4 was first used to examine whether T2 psychological distress statistically mediated the association between T1 physical activity habit automaticity and T3 quality of life. In the baseline-adjusted model (Table 5), T1 physical activity habit automaticity was negatively associated with T2 psychological distress, and T2 psychological distress was negatively associated with T3 quality of life. The indirect effect was significant, and the direct effect from T1 habit automaticity to T3 quality of life remained significant, indicating partial mediation. The mediated proportion was 30.9% (0.043/0.139), suggesting that approximately one-third of the total association between T1 habit automaticity and T3 quality of life was statistically accounted for by T2 psychological distress. The baseline-adjusted longitudinal mediation model is presented in Figure 1.

### 3.5. Moderated Mediation Analysis

PROCESS Model 7 was then used to test whether exercise identity moderated the first-stage path from T1 physical activity habit automaticity to T2 psychological distress. In the baseline-adjusted model (Table 6), the interaction between physical activity habit automaticity and exercise identity significantly predicted T2 psychological distress. The interaction added a small but statistically significant amount of explained variance (ΔR^2^ = 0.008). Thus, the moderation effect was significant but small in magnitude. The pattern of conditional effects in Table 7 indicates that the negative association between habit automaticity and later psychological distress was weak and not statistically significant at low exercise identity but became significant and stronger at mean and high levels of exercise identity. Conditional mediated proportions were 12.2% at low exercise identity, 28.8% at mean exercise identity, and 45.3% at high exercise identity; because the low-identity conditional indirect effect included zero, this value should be interpreted descriptively rather than as evidence of a significant mediated pathway.

**Figure 2:**
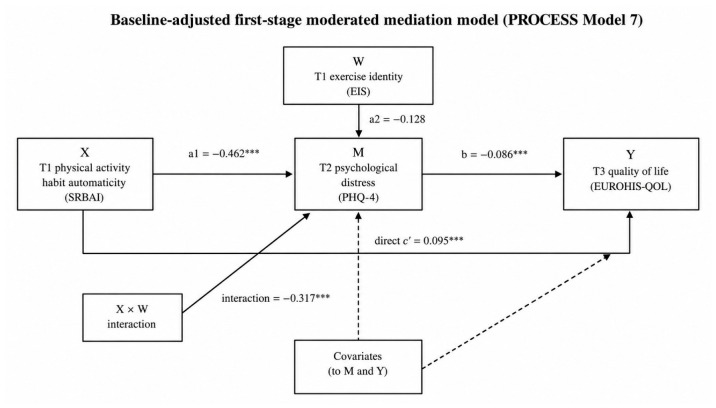
Baseline-adjusted first-stage moderated mediation model (PROCESS Model 7). Note. X = T1 physical activity habit automaticity; W = T1 exercise identity; M = T2 psychological distress; Y = T3 quality of life. Solid arrows indicate hypothesized direct paths; dashed arrows indicate the moderation and covariate-adjustment components. Covariates were entered into the mediator and outcome equations. Model coefficients are reported in Table 6, and conditional indirect effects are reported in Table 7. *** *p* < 0.001.

The hypothesized and estimated first-stage moderated mediation model is presented in Figure 2.

**Figure 3:**
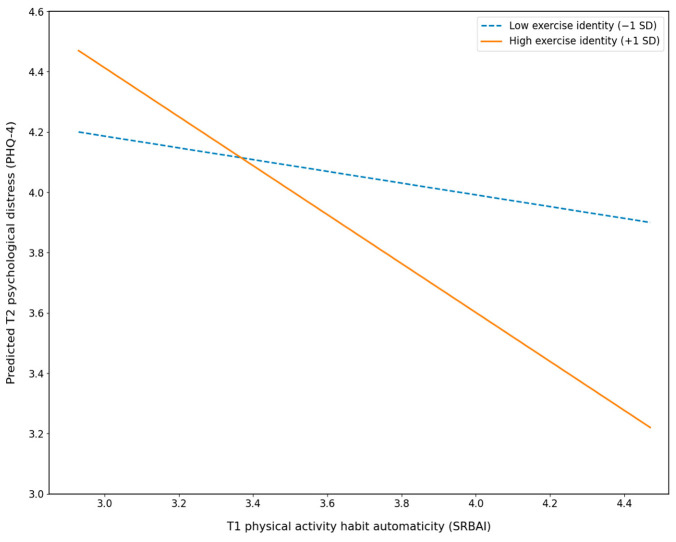
Simple slopes of T1 physical activity habit automaticity predicting T2 psychological distress at low and high levels of exercise identity. Note. Lines represent predicted values calculated from the baseline-adjusted regression equation at T1 habit automaticity mean ± 1 SD and T1 exercise identity mean ± 1 SD, with covariates held at their sample means. The low-identity slope was weaker and not statistically significant (B = −0.197, *p* = 0.088), whereas the high-identity slope was significant and stronger (B = −0.726, *p* < 0.001).

The simple slope pattern for the interaction between physical activity habit automaticity and exercise identity is presented in Figure 3.

## 4. Discussion

This three-wave longitudinal study examined whether physical activity habit automaticity was associated with later quality of life via psychological distress and whether this indirect association depended on exercise identity. The findings generally supported the proposed hypotheses. H1 was supported because T1 physical activity habit automaticity was positively associated with T3 quality of life. H2 was supported because T1 physical activity habit automaticity was negatively associated with T2 psychological distress. H3 was supported because T2 psychological distress significantly mediated the association between T1 habit automaticity and T3 quality of life. H4 was supported because exercise identity significantly moderated the first-stage path from habit automaticity to psychological distress. H5 was partially supported because the index of moderated mediation was significant and the conditional indirect effect was significant at mean and high levels of exercise identity but not at low exercise identity.

The negative association between physical activity habit automaticity and subsequent psychological distress is consistent with the view that health behavior maintenance depends not only on deliberate intention but also on cue-driven automaticity. Previous physical activity research has frequently emphasized volume, frequency, or intensity, whereas the present study focused on whether physical activity had become easy to initiate under familiar cues. This distinction is important because two students may report similar weekly activity, but the student with stronger automaticity may experience less motivational conflict when academic or social demands increase. This finding also extends recent moderated mediation studies in student health in which physical activity was modeled as a moderator or protective behavior in anxiety, sleep hygiene, maltreatment, and depressive-symptom pathways [53,54]. The present study therefore shifts the focus from how often students are active to how easily physical activity is integrated into everyday routines.

The indirect role of psychological distress is also consistent with the previously introduced literature linking physical activity, emotional symptoms, and quality of life. In the present study, lower T2 distress partly accounted for the association between T1 habit automaticity and T3 quality of life after adjustment for baseline quality of life and baseline distress. Similar conditional-process research in student samples has treated rumination or mobile phone addiction as proximal mechanisms linking stressors to burnout or sleep-related functioning [55,56]. Other student health models have likewise placed anxiety in an intermediary position while examining physical activity as a moderating factor [57]. Together, these comparisons suggest that psychological distress may be one meaningful emotional pathway through which automated physical activity relates to later quality of life. At the same time, the persistence of a significant direct effect indicates that other mechanisms, such as sleep, self-efficacy, social participation, or perceived physical functioning, may also contribute to this association.

Exercise identity statistically moderated the first-stage path, although the additional explained variance was small. The association between habit automaticity and lower psychological distress was weakest among students with lower exercise identity and strongest among those with higher exercise identity. This pattern aligns with the identity-based reasoning introduced above: when students view exercise as part of who they are, habitual physical activity may be experienced not only as routine but also as self-consistent behavior. Comparable three-wave and moderated mediation designs in education and behavioral health show that individual resources can shape the translation of earlier predictors into later psychological or behavioral outcomes [58,59]. Recent student health and educational technology studies also illustrate how conditional process models can clarify boundary conditions in longitudinal or temporally ordered designs [60,61]. The present study adds to that literature by identifying exercise identity as a modest boundary condition for the distress-related pathway linking physical activity habit automaticity with quality of life. Nevertheless, the small interaction effect suggests that exercise identity should be treated as a modest boundary condition rather than as a dominant protective factor.

The present study analyzed male and female students simultaneously because the primary aim was to test a general longitudinal conditional process model rather than sex-specific mechanisms. Sex was included as a covariate in all mediation and moderated mediation models to reduce potential confounding. In the descriptive and correlational analyses, sex showed only weak associations with the main study variables, suggesting that sex-related differences were present but not strong enough to warrant sex-stratified modeling as the primary analytic strategy. Nevertheless, sex differences in physical activity, psychological distress, and quality of life remain theoretically important. Future studies with sex-balanced samples should test whether the observed mediation and moderated mediation pathways are invariant across male and female students.

The practical implications should be stated carefully. The results do not show that increasing habit automaticity will necessarily cause improvements in quality of life. They do suggest, however, that campus health promotion may benefit from combining habit-formation strategies with identity-supportive approaches. Instead of relying only on exhortations to exercise more, universities might help students build stable activity cues, reduce barriers to routine physical activity, and foster a non-competitive sense of being an active person. Such strategies could be integrated into physical education, student counseling, residence-life programming, and campus health services. Because the moderation effect was small, identity-based strategies should be viewed as one component of broader student mental health promotion rather than as a stand-alone solution.

Several limitations need to be acknowledged. First, all variables were self-reported, which may introduce recall bias and shared method variance, although the three-wave design and Harman’s single-factor result reduce this concern. Second, the study used observational data, and statistical mediation should not be interpreted as proof of causal pathways. Classical mediation frameworks and contemporary causal inference perspectives both caution against causal overinterpretation when variables are not experimentally manipulated [62,63]. More formal causal mediation approaches require assumptions and designs beyond those available in this study [64,65]. Third, the sample consisted of Chinese university students from a convenience sampling framework, limiting generalizability. Fourth, physical activity frequency was assessed with one item rather than an objective device or full metabolic-equivalent questionnaire. Fifth, although baseline distress and baseline quality of life were controlled, unmeasured factors such as socioeconomic status, academic stress, and social support may still confound the associations.

Future research should move beyond the present design in several ways. First, preregistered longitudinal studies with longer follow-up intervals could clarify whether habit automaticity predicts sustained improvements in emotional symptoms and quality of life over semesters or academic years. Second, objective or multi-method physical activity assessment would help separate the effects of activity volume from the effects of automaticity. Third, intervention studies could test whether cue planning, context stabilization, and identity-supportive messages strengthen physical activity habits and improve student well-being. Fourth, future work should examine additional mediators, such as sleep quality, perceived stress, self-efficacy, and social connectedness, and should test whether the conditional process differs by sex, grade, or baseline mental health risk. Reporting should remain consistent with observational research standards [66,67].

## 5. Conclusions

Among Chinese university students, physical activity habit automaticity was prospectively associated with better quality of life partly through lower psychological distress. Exercise identity statistically, but modestly, moderated the first-stage association between habit automaticity and psychological distress, and the conditional indirect association was significant at mean and high levels of exercise identity. These findings support a temporally separated conditional process model linking automated physical activity, emotional symptom burden, and subsequent quality of life, but they should not be interpreted as evidence of causal effects.

The main advance of this study is that it shifts attention from physical activity frequency alone to the automaticity of physical activity habits. By integrating habit automaticity, psychological distress, exercise identity, and quality of life within a three-wave longitudinal framework, the study provides a more nuanced account of how sustainable physical activity routines may be related to students’ later well-being. The moderated mediation pattern further suggests that the psychological relevance of physical activity habits may be stronger when students also define themselves as exercisers.

From a practical perspective, campus health promotion programs should not focus only on encouraging students to exercise more frequently. Universities may also help students build stable physical activity cues, create predictable exercise routines, reduce barriers to regular participation, and support a positive but non-competitive exercise identity. These strategies may be incorporated into physical education courses, student counseling services, residence-life programs, and campus health initiatives. Because the present study was observational and the moderation effect was small, these applications should be interpreted as directions for health promotion and future intervention research rather than as proof of causal intervention effects.

## Figures and Tables

**Table 1:** Sample flow and longitudinal matching.

Item	*n*
T1 returned questionnaires	1532
T1 initially valid questionnaires	1351
T1 records eligible for matching in the harmonized analytic file	1282
T2 returned questionnaires	1444
T2 valid questionnaires	1329
T3 returned questionnaires	1270
T3 valid questionnaires	1089
Final three-wave matched analytic sample	1024

Note. *n* indicates the number of questionnaires or participants at each screening and matching step. The final analytic sample consisted of students who provided valid and matchable data across all three waves.

**Table 2:** Participant characteristics in the final analytic sample (N = 1024).

Variable	Category/Statistic	Value
Sex	Male	420 (41.02%)
	Female	604 (58.98%)
Age	Mean ± SD	19.93 ± 1.60
	Range	18–27
Grade	First year	324 (31.64%)
	Second year	283 (27.64%)
	Third year	246 (24.02%)
	Fourth year	122 (11.91%)
	Postgraduate	49 (4.79%)
Physical activity frequency	Mean ± SD	2.63 ± 1.46 days/week
Sleep quality	Mean ± SD	3.29 ± 0.77

**Table 3:** Descriptive statistics, distributional indices, and internal consistency.

Variable	M	SD	Min	Max	Skew	Kurtosis	α
Age	19.93	1.60	18.00	27.00	1.76	4.83	-
Grade	2.31	1.17	1.00	5.00	0.55	−0.61	-
Physical activity frequency	2.63	1.46	0.00	7.00	0.19	−0.34	-
Sleep quality	3.29	0.77	1.00	5.00	0.02	−0.16	-
T1 physical activity habit automaticity	3.69	0.85	1.50	6.75	0.05	−0.33	0.833
T1 exercise identity	4.28	0.83	1.56	7.00	0.01	−0.19	0.932
T1 psychological distress	5.03	2.96	0.00	12.00	0.16	−0.59	0.863
T2 psychological distress	3.93	2.66	0.00	12.00	0.39	−0.45	0.834
T1 quality of life	3.56	0.71	1.50	5.00	−0.05	−0.37	0.916
T3 quality of life	3.44	0.72	1.12	5.00	−0.03	−0.29	0.923

Note. SRBAI and EIS were scored as item means. PHQ-4 was scored as a total score. EUROHIS-QOL was scored as an item mean. α = Cronbach’s alpha.

**Table 4:** Pearson correlations among study variables.

Variable	1	2	3	4	5	6	7	8	9	10	11
1. Sex (male = 1)	-										
2. Age	−0.018	-									
3. Grade	−0.016	0.882 ***	-								
4. PA frequency	0.078 *	0.011	0.018	-							
5. Sleep quality	0.006	−0.009	−0.019	0.081 **	-						
6. SRBAI	0.111 ***	0.032	0.064 *	0.401 ***	0.177 ***	-					
7. EIS	0.050	0.073 *	0.079 *	0.383 ***	0.159 ***	0.486 ***	-				
8. T1 PHQ-4	−0.106 ***	−0.014	−0.001	−0.177 ***	−0.390 ***	−0.327 ***	−0.305 ***	-			
9. T2 PHQ-4	−0.098 **	−0.019	−0.011	−0.208 ***	−0.300 ***	−0.367 ***	−0.296 ***	0.605 ***	-		
10. T1 QOL	0.073 *	0.052	0.048	0.164 ***	0.226 ***	0.309 ***	0.230 ***	−0.539 ***	−0.390 ***	-	
11. T3 QOL	0.070 *	0.031	0.011	0.211 ***	0.273 ***	0.405 ***	0.283 ***	−0.591 ***	−0.606 ***	0.675 ***	-

Note. PA = physical activity; SRBAI = Self-Report Behavioral Automaticity Index; EIS = Exercise Identity Scale; PHQ-4 = Patient Health Questionnaire-4; QOL = quality of life. * *p* < 0.05, ** *p* < 0.01, *** *p* < 0.001.

**Table 5:** Baseline-adjusted longitudinal mediation model (PROCESS Model 4).

Outcome/Effect	Predictor/Path	Coefficient/Effect	SE	*p*	95% CI
T2 psychological distress	T1 SRBAI	−0.503	0.088	<0.001	[−0.675, −0.331]
T3 quality of life	T2 psychological distress	−0.086	0.007	<0.001	[−0.099, −0.072]
T3 quality of life	T1 SRBAI direct effect	0.095	0.020	<0.001	[0.057, 0.134]
T3 quality of life	T1 SRBAI total effect	0.139	0.021	<0.001	[0.098, 0.179]
Indirect effect	SRBAI → PHQ-4 → QOL	0.043	-	-	[0.028, 0.059]

Note. Covariates were sex, age, grade, physical activity frequency, sleep quality, T1 psychological distress, and T1 quality of life. Bootstrap confidence intervals were based on 5000 resamples. Coefficients are unstandardized regression coefficients.

**Table 6:** Baseline-adjusted moderated mediation model (PROCESS Model 7).

Outcome	Predictor	B	SE	t	*p*	95% CI
T2 psychological distress	T1 habit automaticity	−0.462	0.092	−4.999	<0.001	[−0.643, −0.281]
T2 psychological distress	T1 exercise identity	−0.128	0.092	−1.393	0.164	[−0.308, 0.052]
T2 psychological distress	T1 habit automaticity × T1 exercise identity	−0.317	0.084	−3.782	<0.001	[−0.482, −0.153]
T3 quality of life	T1 habit automaticity	0.095	0.020	4.879	<0.001	[0.057, 0.134]
T3 quality of life	T2 PHQ-4	−0.086	0.007	−12.48	<0.001	[−0.099, −0.072]

Note. Continuous predictors were mean-centered before forming the interaction term. Covariates were sex, age, grade, physical activity frequency, sleep quality, T1 psychological distress, and T1 quality of life. M-equation R^2^ = 0.414; interaction ΔR^2^ = 0.008. Y-equation R^2^ = 0.615. B = unstandardized coefficient.

**Table 7:** Conditional effects and conditional indirect effects by exercise identity.

Effect	Path	Coefficient/Effect	SE/BootSE	*p*	95% CI
Simple slope: low EIS (−1 SD)	T1 habit automaticity → T2 psychological distress	−0.197	0.115	0.088	[−0.424, 0.029]
Simple slope: mean EIS	T1 habit automaticity → T2 psychological distress	−0.462	0.092	<0.001	[−0.643, −0.281]
Simple slope: high EIS (+1 SD)	T1 habit automaticity → T2 psychological distress	−0.726	0.116	<0.001	[−0.955, −0.498]
Indirect effect: low EIS (−1 SD)	T1 habit automaticity → T2 psychological distress → T3 quality of life	0.017	0.010	-	[−0.003, 0.038]
Indirect effect: mean EIS	T1 habit automaticity → T2 psychological distress → T3 quality of life	0.040	0.008	-	[0.024, 0.057]
Indirect effect: high EIS (+1 SD)	T1 habit automaticity → T2 psychological distress → T3 quality of life	0.063	0.011	-	[0.043, 0.084]
Index of moderated mediation	Conditional indirect association	0.027	0.008	-	[0.012, 0.043]

Note. Simple slopes are unstandardized coefficients from the baseline-adjusted first-stage model. Conditional indirect effects and the index of moderated mediation were estimated with 5000 bootstrap samples. The low-identity indirect effect included zero, whereas the mean- and high-identity indirect effects did not include zero.

## Data Availability

The datasets generated and analyzed during the current study are not publicly available due to privacy and ethical restrictions. Data are available from the corresponding author upon reasonable request and with approval from the corresponding author.

## Abbreviations

EIS, Exercise Identity Scale; EUROHIS-QOL, EUROHIS-QOL 8-item index; PHQ-4, Patient Health Questionnaire-4; QOL, quality of life; SRBAI, Self-Report Behavioral Automaticity Index.
